# Chronic Expanding Hematoma of the Postmastectomy Axilla: A Pictorial Review of Long-Term Multimodality Imaging

**DOI:** 10.7759/cureus.114423

**Published:** 2026-08-12

**Authors:** Katherine Wong, Chun Hin So

**Affiliations:** 1 Radiology, North District Hospital, Hong Kong, HKG; 2 Surgery, Pok Oi Hospital, Hong Kong, HKG

**Keywords:** breast and gynecological pathology, breast lesions and imaging modality, breast screening, chronic encapsulated expanding hematoma, multimodality correlation

## Abstract

Chronic expanding hematomas (CEHs) are rare, benign lesions that can persist and slowly grow following surgery or trauma. In the breast and axillary region, they may closely mimic recurrent malignancy because they may demonstrate mixed internal structures, calcifications, peripheral enhancement, and sometimes even fluorodeoxyglucose (FDG) uptake on positron emission tomography (PET) scans. In this article, we present a long-term imaging review of a patient with a chronic left axillary lesion after bilateral mastectomy, with serial ultrasound, PET/computed tomography (CT), and magnetic resonance imaging (MRI) images and repeated histopathology over nearly two decades. The lesion evolved from a large cystic postoperative collection into a heterogeneous multilobulated mass with mixed solid and cystic components. It demonstrated mild vascularity, wall thickening, and peripheral calcification while remaining pathologically benign on repeated tissue sampling. This pictorial review highlights the imaging evolution, pitfalls, and key differentiating features that help distinguish CEH from recurrent tumors and other postoperative mimics.

## Introduction

Breast hematomas are common after trauma, surgery, biopsy, or antithrombotic treatment, but their imaging appearance may be misleading when they persist or organize over time [1,2]. A chronic expanding hematoma (CEH) is a particular subtype in which the lesion continues to enlarge rather than resolve, often because of repeated bleeding, inflammation, fibrous capsule formation, and organization of a clot [3,4]. Breast hematomas were first described by Reid et al., with the authors defining CEHs as hematomas that increase in size for more than one month after the causative event [5,6]. Although rare, CEHs are clinically important because they can simulate recurrent carcinoma, chest wall tumor, abscess, fat necrosis, or a postoperative seroma [4-6].

CEH is well recognized in postsurgical soft tissues and has been reported in the breast after explantation or reconstruction, where magnetic resonance imaging (MRI) often plays a major role in diagnosis [2,3,6]. This pictorial review uses one long-term case to demonstrate the imaging spectrum of a chronic postoperative axillary hematoma extending to the subpectoral chest wall. The key teaching point is that temporal evolution and pathology correlation are often more useful than any single snapshot image [4,5].

## Case presentation

**Figure 1:**
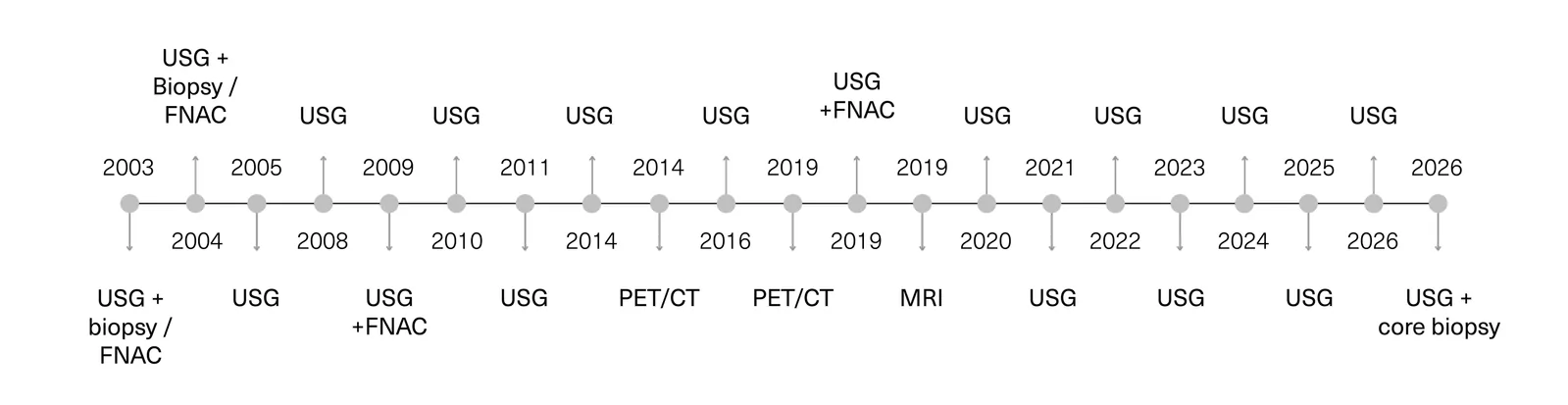
Chronological timeline of multimodality imaging and tissue biopsies performed from 2003 to 2026, demonstrating the evolution of the left axillary/chest wall lesion. Chronological summary of the left axillary/chest wall lesion over more than two decades (2003-2026), including serial imaging studies (USG, PET/CT, MRI) and tissue sampling procedures (FNA and core biopsy). USG, ultrasound; PET/CT, positron emission tomography/computed tomography; MRI, magnetic resonance imaging; FNA, fine-needle aspiration; FNAC, fine-needle aspiration cytology

The patient had a history of bilateral mastectomy with postoperative scarring in the bilateral anterior chest wall and axillae. A left axillary hematoma was first identified on ultrasound in 2005, and fine-needle aspiration cytology (FNAC) yielded blood only. The lesion decreased in size on follow-up in 2008, then increased again by 2009 to about 6 cm. Positron emission tomography/computed tomography (PET/CT) in 2016 showed a non-fluorodeoxyglucose (FDG)-avid left axillary seroma with faint rim uptake. PET/CT in 2019 demonstrated a hypodense left axillary collection with FDG-avid foci along the rim and subcutaneous nodules. The MRI in 2019 favored a CEH with possible superimposed inflammation. Multiple subsequent biopsies and FNACs in 2005, 2009, 2019, and 2023 showed blood, necrotic tissue, or inflammatory change without malignancy (Figure 1).

Ultrasound evolution

**Figure 2:**
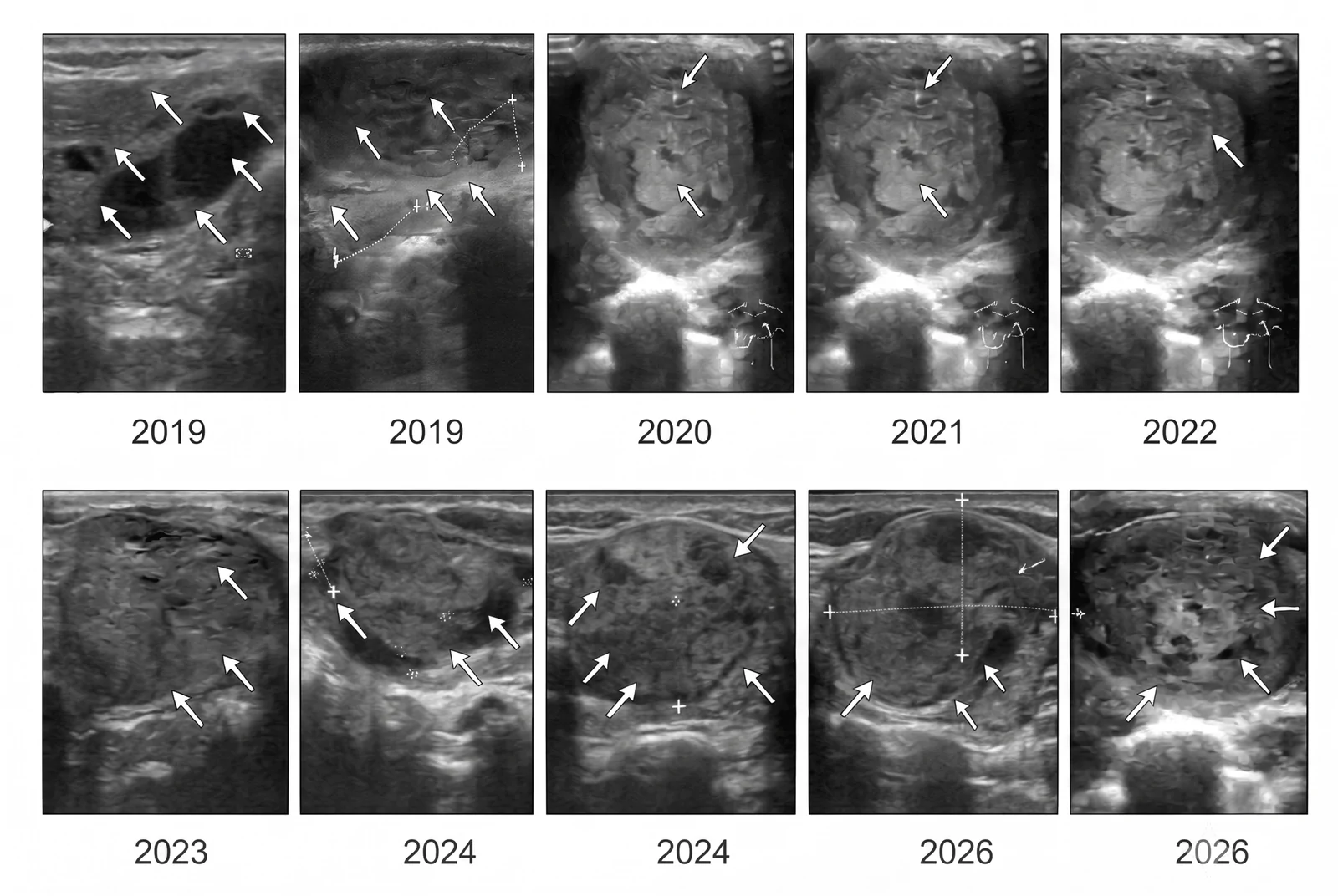
Serial USG images demonstrating the longitudinal progression and morphological changes of the left axillary/chest wall lesion from 2019 to 2026. Chronological USG series spanning seven years (2019-2026) detailing the gradual enlargement and structural evolution of the left axillary/chest wall mass. The arrows indicate the boundaries and internal features of the lesion, showing progressive changes from initial fluid-debris components and thick-walled collections to a larger heterogeneous mass with increased solid components. USG, ultrasound

In this case, the earliest lesion was mostly hematoma-like and yielded blood on aspiration, but later scans showed gradual enlargement and increasing internal complexity. Chronological ultrasound series spanning seven years (2019-2026) detailed the gradual enlargement and structural evolution of the left axillary/chest wall mass (Figure 2). The most recent ultrasound on 25 March 2026 demonstrated a large heterogeneous multilobulated mass in the left axilla extending medially along the upper lateral anterior chest wall at the subpectoralis level.

In 2003, ultrasound showed a dense, microlobulated, circumscribed suspicious mass in the left axillary tail measuring 1.6 x 2.4 x 2.1 cm, with a solitary granular microcalcification. In 2005, the lesion had enlarged into a thick-walled collection with internal echoes, measuring approximately 9.6 x 2.9 x 8.2 cm, consistent with an organized hematoma; importantly, there was no wall vascularity, and FNA yielded old blood. In 2006, the lesion was described as a benign organized hematoma/seroma, and in 2008, it had decreased to about 2.7 x 2.0 x 0.8 cm, with no significant interval change. In 2009, it increased again to about 6.0 x 2.6 x 1.0 cm, and FNAC yielded altered blood; the lesion then remained relatively stable in 2010 (3.4 x 1.3 x 6.0 cm) and 2011 (3.2 x 1.0 cm transverse diameter). By 2014, the lesion still appeared as a thick-walled organized hematoma but with increasing cystic areas compatible with hemolysis. In 2020, ultrasound showed a longer craniocaudal span of 14.24 cm with a thick wall, heterogeneous internal echoes, coarse calcifications, and a tiny central fluid component, suggesting further organization. In 2021, the lesion remained largely similar, measuring 6.32 x 1.8 cm in the main bulk and 10.8 cm in craniocaudal extent. In 2022, it was slightly larger at about 7.6 x 2.0 x 3.0 cm. In 2023, a new discrete hypoechoic lobulated mass deep to the pectoralis major appeared, measuring 2.9 x 1.5 x 1.5 cm, with increased internal vascularity and suspicious features. In 2024, the mass was grossly similar but with a suspicious superolateral soft tissue component and a thick wall. In 2025, the superolateral hypoechoic lobulated mass had enlarged to 4.4 x 4.1 x 4.6 cm, and by 2026, the lesion had become a large heterogeneous multilobulated mass measuring about 6.1 x 3.8 x 11.6 cm, with mild internal vascularity, calcified foci, thinning of overlying skin, and a decreasing cystic component over time (Figure 2).

CT and PET/CT findings

**Figure 3:**
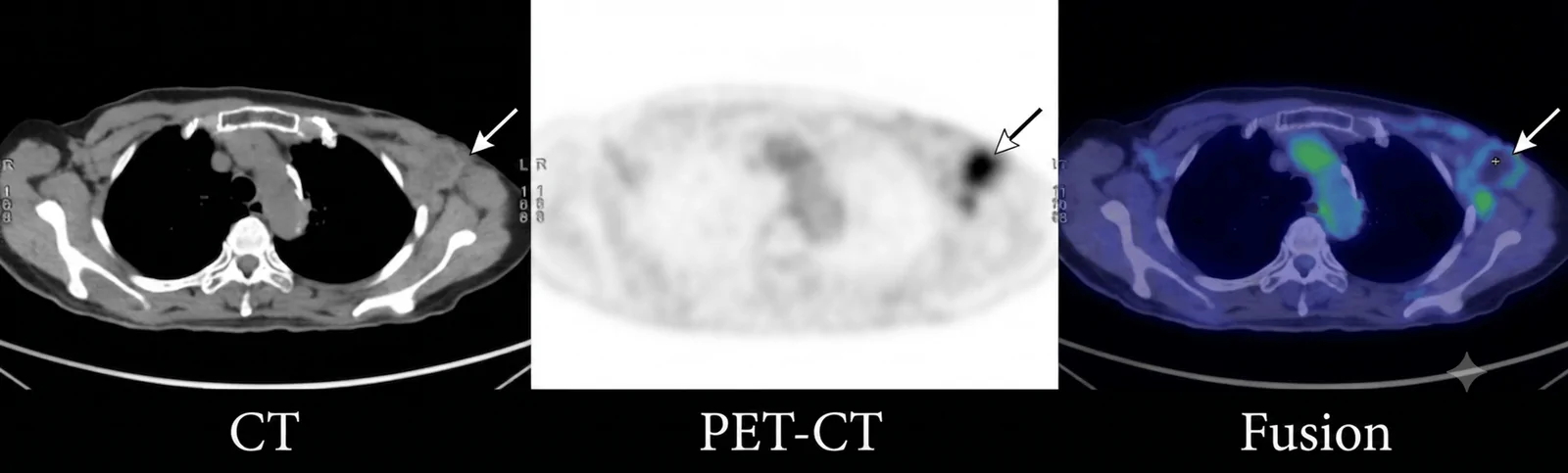
Axial CT, PET, and fused PET-CT images demonstrating the left axillary/chest wall lesion. Axial cross-sectional imaging of the left axillary/chest wall region displaying the lesion via noncontrast CT, PET, and fused PET-CT modalities. White arrows indicate the location of the axillary mass, highlighting its anatomical and metabolic characteristics. CT, computed tomography; PET, positron emission tomography

On CT and PET/CT, the lesion persisted as a left axillary seroma/collection with chronic postoperative change, showing a hypodense fluid component and only faint to mild peripheral rim FDG uptake, with later studies demonstrating small hypermetabolic rim foci rather than a frankly solid, intensely avid mass (Figure 3). This pattern is more in keeping with a CEH or organized postoperative collection than with aggressive recurrence, particularly when the uptake is peripheral, and the lesion remains largely cystic or mixed-density over time.

MRI findings

**Figure 4:**
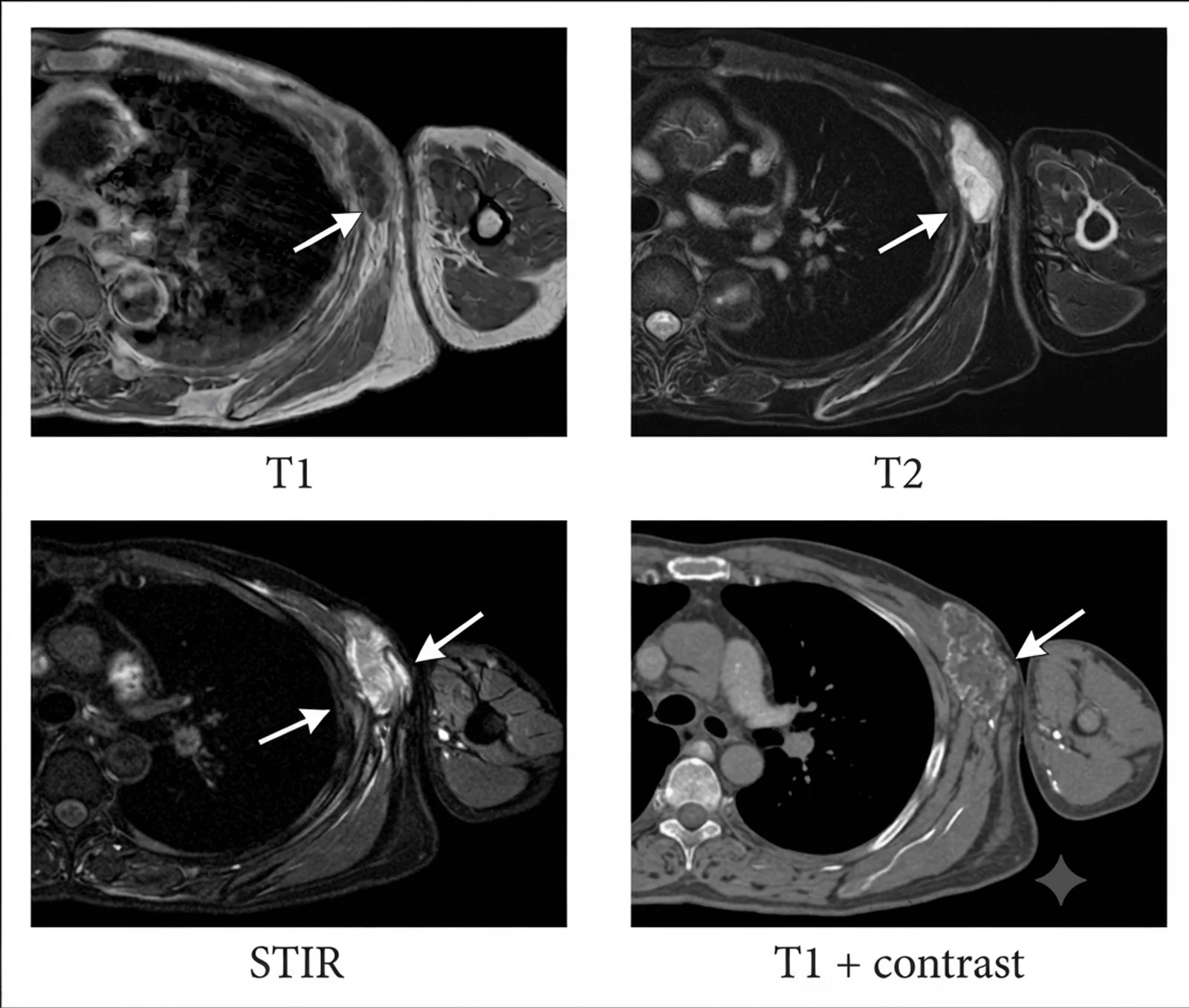
Axial MRI sequences of the left axilla demonstrating the left axillary/chest wall lesion. Axial MRI sequences presented in a four-panel grid: T1-weighted (T1), T2-weighted (T2), STIR, and post-contrast T1-weighted (T1+C) sequences. The white arrows indicate a mass located in the left axillary/chest wall, which appears heterogeneously hyperintense on T2 and STIR images and shows heterogeneous enhancement after gadolinium administration. MRI, magnetic resonance imaging; STIR, short tau inversion recovery

MRI in 2019 showed a heterogeneous lesion in the left upper lateral anterior chest wall extending to the axillary level, measuring 3.4 x 3.3 x 11.2 cm, with mixed T1 iso- to hyperintense and T2 heterogeneous iso- to hyperintense signal, in keeping with a chronic organized hematoma/CEH rather than a simple fluid collection (Figure 4).

## Discussion

Imaging findings

Ultrasound is often the first modality to detect a postoperative breast or axillary collection. Acute hematomas are typically more cystic or complex fluid-filled, while chronic lesions progressively become heterogeneous due to clot breakdown, organizing debris, and fibrosis [1,2]. A typical chronic or organized hematoma on ultrasound is usually a well-defined hypoechoic or mixed-echogenicity mass with internal echoes/debris, possible capsule or thick wall, and absent internal Doppler flow; a thin peripheral vascular rim may be reactive. Over time, it may become more complex rather than simpler, with septations, fluid-debris levels, peripheral calcifications, and echogenic fibrous bands, which can make it difficult to distinguish from a tumor [7].

On CT, CEHs are typically seen as well-circumscribed, low-attenuation masses that may contain internal septations, mixed soft-tissue density, or peripheral capsule enhancement, while PET/CT may show absent, faint, or peripheral FDG uptake from inflammation or granulation tissue rather than uniform avidity. In a pictorial review, the most useful CT/PET-CT images to show are the non-avid seroma phase, the faint rim-avid phase, and the later rim hypermetabolic phase, because together they illustrate the slow evolution from a postoperative collection toward a CEH that can mimic a tumor [7].

On PET-CT, it is important to note that FDG uptake does not automatically imply cancer in postoperative lesions. Inflammation, reparative tissue, and granulation tissue can all be metabolically active [5,7]. Therefore, PET/CT findings should always be interpreted together with chronology, morphology, and prior pathology [4,5].

On MRI, CEH commonly shows a well-defined or multilobulated mass with a fibrous pseudocapsule, internal signal heterogeneity from blood products of different ages, and variable T1/T2 signal depending on methemoglobin, clot organization, and liquefaction; a peripheral low-signal rim from hemosiderin or fibrosis is also common. By contrast, soft tissue neoplasms are more likely to show a true enhancing solid component, infiltrative margins, and progressive internal nodular enhancement rather than only peripheral capsule enhancement, although overlap is substantial and biopsy is often needed when imaging is indeterminate. A CEH may look tumor-like on MRI, but the combination of long-term persistence, prior surgery or bleeding, mixed blood-product signal, and repeated negative cytology/biopsy favors a benign organizing hematoma rather than malignancy [3].

Pathology correlation

When imaging findings remain indeterminate, repeat FNAC and biopsy are essential for further evaluation. In this case, serial aspiration and biopsy specimens obtained over several years consistently demonstrated blood, necrotic debris, and inflammatory changes, without evidence of malignant cells. This repeated benign histology strongly supports the diagnosis of a CEH. Chronic breast hematomas may also be associated with fat necrosis, fibrosis, and chronic inflammatory changes, which can contribute to the presence of a firm palpable mass and the complex internal appearance on imaging [1,2].

In this setting, pathology serves not only to exclude malignancy but also to clarify why a lesion may behave clinically and radiologically like a neoplasm despite being benign. The diagnosis is most convincing when the pathological and imaging findings remain concordant over time [1,2].

Differential diagnosis

The main differential diagnoses for a chronic postoperative axillary mass include recurrent breast carcinoma, postoperative seroma, abscess, fat necrosis, foreign-body reaction, and, less commonly, a second tumor arising in a chronic collection [2,3,7,8]. Recurrence is usually favored by progressive infiltration, irregular enhancing soft tissue, and interval nodal or distant disease. An abscess is suggested by pain, systemic signs, and thick enhancing walls with surrounding edema. Fat necrosis often shows oil cysts, calcifications, and a more typical postoperative evolution, while chronic organizing hematoma tends to have a long, stepwise course with repeated benign sampling [1,2,6,8].

In this case, the preserved axillary vessels, lack of definite infiltration, mixed cystic-solid architecture, calcified foci, and very long history after mastectomy made CEH the most plausible diagnosis.

## Conclusions

CEH of the post-mastectomy axilla is a rare but important benign mimic of recurrent malignancy. It may evolve over many years from a cystic postoperative collection into a heterogeneous multilobulated lesion with calcification, mild vascularity, wall thickening, and even FDG uptake. The strongest diagnostic clue is the long-term imaging history, especially when repeated biopsies show only blood, necrotic tissue, or inflammation. This pictorial review demonstrates that multidisciplinary correlation and careful review of prior studies are essential to avoid overtreatment and to recognize this unusual but characteristic postoperative entity.
